# Diacetamidinium sulfate

**DOI:** 10.1107/S1600536810049160

**Published:** 2010-11-27

**Authors:** Zdeněk Jalový, Aleš Růžička

**Affiliations:** a Institute of Energetic Materials, Faculty of Chemical Technology, University of Pardubice, Studentská 573, Pardubice 532 10, Czech Republic; b Department of General and Inorganic Chemistry, Faculty of Chemical Technology, University of Pardubice, Studentská 573, Pardubice 532 10, Czech Republic

## Abstract

In the crystal structure of the title compound, 2C_2_H_7_N_2_^+^·SO_4_^2−^, which contains four cations and two anions in the asymmetric unit, the ions are inter­connected by an extensive hydrogen-bonding system whereby two of the O atoms of sulfate ion are hydrogen-bonded to the amidinium H atoms of two cations, leading to the formation of two eight-membered rings. The two remaining O atoms inter­connect two H atoms of acetamidinium cations, forming an infinite chain. The C⋯N separations within the H_2_N⋯C⋯NH_2_ moieties are similar, with an average value of 1.305 (2) Å, which is in good agreement with a delocalization model.

## Related literature

For preparation, reactivity and behaviour of similar compounds, see: Jalový *et al.* (2005 ▶); Latypov *et al.* (1998 ▶); Taylor & Ehrhart (1960 ▶). For related structures, see: Calov & Jost (1990 ▶); Cannon *et al.* (1976 ▶); Emirdag-Eanes & Ibers (2002) ▶; Ferretti *et al.* (2004 ▶); Jalový *et al.* (2009 ▶); Tominey *et al.* (2006 ▶).
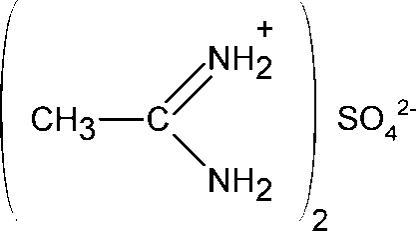


## Experimental

### 

#### Crystal data

2C_2_H_7_N_2_^+^·SO_4_^2−^
*M* *_r_* = 214.26
Triclinic, 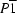

*a* = 8.0961 (3) Å
*b* = 11.1668 (4) Å
*c* = 11.8821 (6) Å
α = 96.199 (4)°
β = 105.905 (3)°
γ = 105.615 (4)°
*V* = 975.63 (8) Å^3^
*Z* = 4
Mo *K*α radiation
μ = 0.33 mm^−1^
*T* = 150 K
0.44 × 0.23 × 0.21 mm

#### Data collection

Bruker–Nonius KappaCCD area-detector diffractometer
Absorption correction: Gaussian (Coppens, 1970 ▶) *T*_min_ = 0.915, *T*_max_ = 0.958
20866 measured reflections
4459 independent reflections
3623 reflections with *I* > 2σ(*I*)
*R*_int_ = 0.040

#### Refinement

*R*[*F* ^2^ > 2σ(*F* ^2^)] = 0.040
*wR*(*F* ^2^) = 0.105
*S* = 1.10
4459 reflections
239 parameters
H-atom parameters constrained
Δρ_max_ = 0.23 e Å^−3^
Δρ_min_ = −0.46 e Å^−3^

### 

Data collection: *COLLECT* (Hooft, 1998 ▶) and *DENZO* (Otwin­owski & Minor, 1997 ▶); cell refinement: *COLLECT* and *DENZO*; data reduction: *COLLECT* and *DENZO*; program(s) used to solve structure: *SIR92* (Altomare *et al.*, 1994 ▶); program(s) used to refine structure: *SHELXL97* (Sheldrick, 2008 ▶); molecular graphics: *PLATON* (Spek, 2009 ▶); software used to prepare material for publication: *SHELXL97*.

## Supplementary Material

Crystal structure: contains datablocks I, global. DOI: 10.1107/S1600536810049160/rk2242sup1.cif

Structure factors: contains datablocks I. DOI: 10.1107/S1600536810049160/rk2242Isup2.hkl

Additional supplementary materials:  crystallographic information; 3D view; checkCIF report

## Figures and Tables

**Table 1:** Hydrogen-bond geometry (Å, °)

*D*—H⋯*A*	*D*—H	H⋯*A*	*D*⋯*A*	*D*—H⋯*A*
N12—H12*A*⋯O1^i^	0.86	2.02	2.838 (2)	158
N12—H12*A*⋯S1^i^	0.86	2.93	3.6038 (17)	136
N12—H12*B*⋯O6^ii^	0.86	1.99	2.843 (2)	172
N12—H12*B*⋯S2^ii^	0.86	2.98	3.7523 (17)	150
N18—H18*A*⋯O8	0.86	1.99	2.826 (2)	164
N18—H18*A*⋯S2	0.86	2.90	3.6006 (17)	140
N18—H18*B*⋯O3	0.86	1.99	2.823 (2)	164
N15—H15*A*⋯O2	0.86	2.03	2.841 (2)	157
N15—H15*A*⋯S1	0.86	2.92	3.5841 (17)	136
N15—H15*B*⋯S2	0.86	3.01	3.7829 (17)	150
N15—H15*B*⋯O5	0.86	1.97	2.817 (2)	169
N16—H16*A*⋯O1	0.86	2.09	2.915 (2)	160
N16—H16*A*⋯S1	0.86	2.85	3.5245 (18)	137
N16—H16*B*⋯O3^iii^	0.86	2.00	2.852 (2)	170
N16—H16*B*⋯S1^iii^	0.86	2.90	3.6821 (17)	152
N11—H11*A*⋯O2^i^	0.86	2.10	2.922 (2)	161
N11—H11*A*⋯S1^i^	0.86	2.84	3.5335 (17)	138
N11—H11*B*⋯O4^iv^	0.86	2.01	2.856 (2)	170
N11—H11*B*⋯S1^iv^	0.86	2.88	3.6657 (17)	152
N13—H13*A*⋯O7^ii^	0.86	1.99	2.826 (2)	165
N13—H13*A*⋯S2^ii^	0.86	2.93	3.6408 (18)	142
N13—H13*B*⋯O4^i^	0.86	1.99	2.835 (2)	165
N14—H14*A*⋯O8^ii^	0.86	2.09	2.938 (2)	167
N14—H14*A*⋯S2^ii^	0.86	2.87	3.5920 (18)	143
N14—H14*B*⋯O6^iv^	0.86	2.02	2.863 (2)	167
N14—H14*B*⋯S2^iv^	0.86	2.96	3.6973 (17)	145
N17—H17*A*⋯O7	0.86	2.12	2.964 (2)	167
N17—H17*B*⋯S2^v^	0.86	2.95	3.7158 (17)	149
N17—H17*B*⋯O5^v^	0.86	2.01	2.861 (2)	168
N17—H17*B*⋯S2^v^	0.86	2.95	3.7158 (17)	149

Symmetry codes: (i) 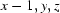; (ii) 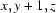; (iii) 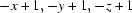; (iv) 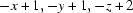; (v) 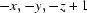.

**Fig. 1.**
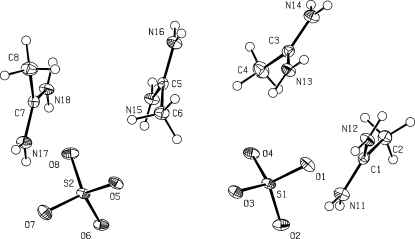
View of the title molecule with the atom numbering scheme. Displacement ellipsoids are shown on 50% probability level. The H atoms are presented as a spheres of arbitrary radius.

**Fig. 2.**
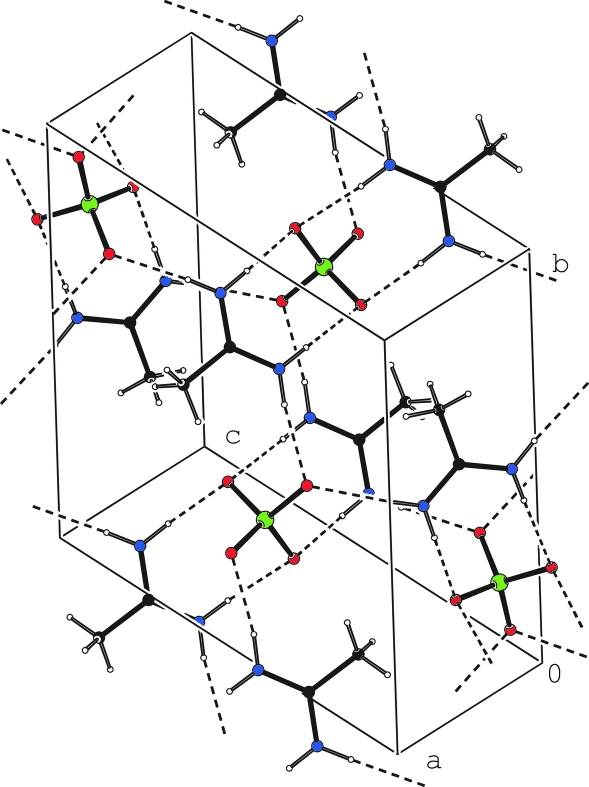
View of the motif of the structure with the hydrogen bonding.

## supplementary crystallographic information

### Comment

Acetamidinium sulphate, C_4_H_14_N_4_O_4_S, (Scheme 1), is a starting material for the synthesis of insensitive explosive 2,2–dinitroethene–1,1–diamine (Latypov *et al.*, 1998; Jalový *et al.*, 2005). It has low hygroscopicity with comparison to commercially available acetamidinium hydrochloride. The title compound was prepared from acetamidinium acetate and equivalent amount of sulfuric acid.

The crystal structure of acetamidinium sulfate has been determined in order to evaluate the degree of association of these ionic species. Two crystallographically independent bisacetamidinium sulfates were found in the unit cell (Fig. 1). The molecular structure of the title compound is made up of two mutually similar acetamidinium units and one sulfate ion. All these ions are interconnected by extensive hydrogen bonding systems where two of the oxygen atoms of sulfate ion (O1 and O2) are bonded to the *exo*–amidinium hydrogen atoms of two units which leading to the formation of two 8–membered rings (Fig. 2). The S–O distances for these particular oxygen atoms O1 and O2 are slightly elongated in comparison to remaining two oxygen atoms O3 and O4  which form a chain. On the other hand, two remaining oxygen atoms interconnect two *endo*–hydrogen atoms of acetamidinium units forming thus an infinite chain. The C–N separations within the H_2_N···C···NH_2_ fragments are mutually similar with average value of 1.305 (2)Å which is with a good agreement with a delocalization concept of the double bond in H_2_N-C(CH_3_)═NH_2_ cationand the literature data, where the range 1.302–1.312Å was found. The comparison of the title compound with the published structures can be made on the bases of two different criteria. The first, all acetamidinium salts reveal the same geometry and structural parameters of the acetamidinium ion. The second criterion is the type of the supramolecular structure formed. There are large differences between the title compound where the two planar layers of acetamidinium ions are interconnected to the infinite double layer. Other 2D structures are found for the acetamidinium formate (Tominey *et al.*, 2006), dinitromethanide (Jalový *et al.*, 2009), amidinium acetate (Ferretti *et al.*, 2004) with the stairs like layered structure and one of the polymorphs of amidinium (2–hydroxyethoxy)acetate (Ferretti *et al.*, 2004). On the other hand, the second polymorph of amidinium (2–hydroxyethoxy)acetate (Ferretti *et al.*, 2004), acetamidinium tetrazolate (Tominey *et al.*, 2006) and acetamidinium chloride (Cannon *et al.*, 1976) reveal 3D structures with large cavities. There are a couple of related acetamidinium ion containing structures which are of interest as for example bis(acetamidinium) hexafluorosilicate (Calov & Jost, 1990) where the multicentered contacts between acetamidinium hydrogen atoms and fluorine atoms were found and the selenium and rhenium containing cluster compound where the hydrogen contacts of acetamidinium ion with cyano group bonded to the rhenium atoms and a short contact between selenium and nitrogen atoms were found (Emirdag-Eanes *et al.*, 2002).

### Experimental

Acetamidinium acetate (1.50 g, 12.7 mmol; Taylor & Ehrhart, 1960) was dissolved in propan–2–ol (15 ml). Sulfuric acid (100%; 0.62 g, 6.3 mmol) was then slowly added. The precipitated product was filtered and washed with fresh propan–2–ol to give 1.15 g (84.6 %) of white solid, m.p. 483–485 K (484-485 K; Jalový *et al.*, 2005). Elementary analysis calc. for C_4_H_14_N_4_O_4_S: C, 22.42%; H, 6.58%; N, 26.16%; S, 14.96%. Found: C, 23.16%; H, 6.27%; N, 25.98%; S, 15.26%. Spectral characteristic are the same as described previously by Jalový *et al.*, (2005). The crystals suitable for X–ray were prepared by crystallization from methanol by slow cooling of the hot solution.

### Refinement

All the hydrogens were discernible in the difference electron density map. However, all the hydrogens were situated into idealized positions and refined riding on their parent C or N atoms, with N—H = 0.86Å, C—H = 0.96Å for methyl, *U*_iso_(H) = 1.2*U*_eq_(N) and *U*_iso_(H) = 1.5*U*_eq_(C) for methyl H atoms, respectively.

### Crystal data

**Table:**

2C_2_H_7_N_2_^+^·O_4_S^2^^−^	*Z* = 4
*M**_r_* = 214.26	*F*(000) = 456
Triclinic, *P*1	*D*_x_ = 1.459 Mg m^−^^3^
Hall symbol: -P 1	Melting point = 483–485 K
*a* = 8.0961 (3) Å	Mo *K*α radiation, λ = 0.71073 Å
*b* = 11.1668 (4) Å	Cell parameters from 20921 reflections
*c* = 11.8821 (6) Å	θ = 1–27.5°
α = 96.199 (4)°	µ = 0.33 mm^−^^1^
β = 105.905 (3)°	*T* = 150 K
γ = 105.615 (4)°	Block, colourless
*V* = 975.63 (8) Å^3^	0.44 × 0.23 × 0.21 mm

### Data collection

**Table:**

Bruker–Nonius KappaCCD area-detector diffractometer	4459 independent reflections
Radiation source: fine–focus sealed tube	3623 reflections with *I* > 2σ(*I*)
graphite	*R*_int_ = 0.040
Detector resolution: 9.091 pixels mm^-1^	θ_max_ = 27.5°, θ_min_ = 2.4°
φ– and ω–scans to fill the Ewald sphere	*h* = −10→10
Absorption correction: gaussian (Coppens, 1970)	*k* = −14→14
*T*_min_ = 0.915, *T*_max_ = 0.958	*l* = −15→15
20866 measured reflections	

### Refinement

**Table:**

Refinement on *F*^2^	Primary atom site location: structure-invariant direct methods
Least-squares matrix: full	Secondary atom site location: difference Fourier map
*R*[*F*^2^ > 2σ(*F*^2^)] = 0.040	Hydrogen site location: inferred from neighbouring sites
*wR*(*F*^2^) = 0.105	H-atom parameters constrained
*S* = 1.10	*w* = 1/[σ^2^(*F*_o_^2^) + (0.0486*P*)^2^ + 0.6492*P*] where *P* = (*F*_o_^2^ + 2*F*_c_^2^)/3
4459 reflections	(Δ/σ)_max_ < 0.001
239 parameters	Δρ_max_ = 0.23 e Å^−^^3^
0 restraints	Δρ_min_ = −0.46 e Å^−^^3^

### Special details

**Table:**

Geometry. All s.u.'s (except the s.u. in the dihedral angle between two l.s. planes) are estimated using the full covariance matrix. The cell s.u.'s are taken into account individually in the estimation of s.u.'s in distances, angles and torsion angles; correlations between s.u.'s in cell parameters are only used when they are defined by crystal symmetry. An approximate (isotropic) treatment of cell s.u.'s is used for estimating s.u.'s involving l.s. planes.
Refinement. Refinement of *F*^2^ against ALL reflections. The weighted *R*–factor w*R* and goodness of fit *S* are based on *F*^2^, conventional *R*–factors *R* are based on *F*, with *F* set to zero for negative *F*^2^. The threshold expression of *F*^2^ > 2σ(*F*^2^) is used only for calculating *R*–factors(gt) etc. and is not relevant to the choice of reflections for refinement. *R*–factors based on *F*^2^ are statistically about twice as large as those based on *F*, and *R*–factors based on ALL data will be even larger.

### Fractional atomic coordinates and isotropic or equivalent isotropic displacement parameters (Å^2^)

**Table:**

	*x*	*y*	*z*	*U*_iso_*/*U*_eq_	
S1	0.79775 (6)	0.46840 (4)	0.72331 (4)	0.01727 (12)	
S2	0.19510 (6)	0.03481 (4)	0.78098 (4)	0.01627 (12)	
O6	0.16125 (19)	0.02135 (13)	0.89534 (12)	0.0213 (3)	
O7	0.1162 (2)	−0.08915 (13)	0.69712 (12)	0.0239 (3)	
O3	0.71625 (18)	0.37497 (12)	0.60982 (12)	0.0209 (3)	
O2	0.71148 (19)	0.42348 (14)	0.81242 (12)	0.0235 (3)	
O4	0.99312 (18)	0.48642 (13)	0.76995 (12)	0.0219 (3)	
O5	0.11266 (18)	0.12598 (12)	0.72686 (12)	0.0208 (3)	
N12	−0.0434 (2)	0.77755 (16)	0.91285 (15)	0.0216 (3)	
H12A	−0.1251	0.7271	0.8509	0.026*	
H12B	0.0103	0.8539	0.9086	0.026*	
O8	0.39298 (18)	0.07972 (14)	0.80283 (12)	0.0240 (3)	
N18	0.4617 (2)	0.13845 (16)	0.59125 (15)	0.0215 (3)	
H18A	0.4545	0.1115	0.6554	0.026*	
H18B	0.5520	0.2017	0.5939	0.026*	
N15	0.3313 (2)	0.36087 (16)	0.70691 (15)	0.0215 (3)	
H15A	0.4408	0.3939	0.7535	0.026*	
H15B	0.2610	0.2951	0.7205	0.026*	
O1	0.7676 (2)	0.59000 (13)	0.70173 (12)	0.0242 (3)	
N16	0.3762 (2)	0.51104 (15)	0.59222 (15)	0.0213 (3)	
H16A	0.4861	0.5455	0.6377	0.026*	
H16B	0.3343	0.5421	0.5315	0.026*	
N11	−0.0796 (2)	0.62260 (16)	1.02350 (15)	0.0215 (3)	
H11A	−0.1618	0.5704	0.9628	0.026*	
H11B	−0.0493	0.5990	1.0907	0.026*	
N13	0.2530 (2)	0.72388 (16)	0.79442 (15)	0.0225 (4)	
H13A	0.2178	0.7767	0.7537	0.027*	
H13B	0.1849	0.6465	0.7801	0.027*	
N14	0.5176 (2)	0.87828 (16)	0.90289 (15)	0.0227 (4)	
H14A	0.4860	0.9332	0.8636	0.027*	
H14B	0.6201	0.9001	0.9585	0.027*	
C7	0.3355 (3)	0.08310 (17)	0.48927 (17)	0.0183 (4)	
N17	0.1957 (2)	−0.01346 (15)	0.48128 (15)	0.0215 (3)	
H17A	0.1850	−0.0424	0.5440	0.026*	
H17B	0.1146	−0.0480	0.4132	0.026*	
C3	0.4094 (3)	0.76088 (18)	0.87780 (17)	0.0195 (4)	
C5	0.2718 (3)	0.41071 (17)	0.61547 (17)	0.0179 (4)	
C1	−0.0009 (3)	0.73845 (18)	1.01366 (17)	0.0182 (4)	
C6	0.0825 (3)	0.3512 (2)	0.53533 (19)	0.0242 (4)	
H6A	0.0713	0.2708	0.4909	0.036*	
H6B	0.0520	0.4056	0.4811	0.036*	
H6C	0.0020	0.3390	0.5822	0.036*	
C4	0.4675 (3)	0.6675 (2)	0.9475 (2)	0.0314 (5)	
H4A	0.4360	0.6735	1.0197	0.047*	
H4B	0.5960	0.6857	0.9670	0.047*	
H4C	0.4079	0.5834	0.9007	0.047*	
C2	0.1430 (3)	0.82735 (19)	1.11939 (18)	0.0235 (4)	
H2A	0.2583	0.8190	1.1205	0.035*	
H2B	0.1189	0.8074	1.1911	0.035*	
H2C	0.1442	0.9128	1.1146	0.035*	
C8	0.3526 (3)	0.1306 (2)	0.37894 (18)	0.0278 (5)	
H8A	0.2818	0.1869	0.3620	0.042*	
H8B	0.3099	0.0602	0.3131	0.042*	
H8C	0.4771	0.1753	0.3907	0.042*	

### Atomic displacement parameters (Å^2^)

**Table:**

	*U*^11^	*U*^22^	*U*^33^	*U*^12^	*U*^13^	*U*^23^
S1	0.0150 (2)	0.0162 (2)	0.0149 (2)	−0.00007 (17)	0.00017 (17)	0.00432 (17)
S2	0.0153 (2)	0.0161 (2)	0.0141 (2)	0.00153 (17)	0.00173 (17)	0.00533 (16)
O6	0.0210 (7)	0.0234 (7)	0.0168 (7)	0.0018 (5)	0.0056 (5)	0.0072 (5)
O7	0.0275 (8)	0.0182 (7)	0.0197 (7)	0.0040 (6)	0.0012 (6)	0.0022 (5)
O3	0.0208 (7)	0.0179 (7)	0.0167 (6)	0.0004 (5)	0.0004 (5)	0.0026 (5)
O2	0.0189 (7)	0.0289 (8)	0.0194 (7)	0.0017 (6)	0.0048 (6)	0.0090 (6)
O4	0.0154 (7)	0.0233 (7)	0.0210 (7)	0.0006 (5)	0.0008 (5)	0.0072 (5)
O5	0.0201 (7)	0.0172 (7)	0.0211 (7)	0.0030 (5)	0.0015 (5)	0.0079 (5)
N12	0.0230 (9)	0.0174 (8)	0.0190 (8)	0.0015 (6)	0.0028 (7)	0.0034 (6)
O8	0.0158 (7)	0.0305 (8)	0.0223 (7)	0.0027 (6)	0.0035 (6)	0.0102 (6)
N18	0.0202 (8)	0.0201 (8)	0.0193 (8)	−0.0008 (6)	0.0059 (7)	0.0036 (6)
N15	0.0186 (8)	0.0202 (8)	0.0233 (8)	0.0024 (6)	0.0052 (7)	0.0083 (7)
O1	0.0267 (8)	0.0163 (7)	0.0222 (7)	0.0029 (6)	−0.0001 (6)	0.0041 (5)
N16	0.0206 (8)	0.0210 (8)	0.0187 (8)	0.0035 (7)	0.0020 (7)	0.0081 (6)
N11	0.0203 (8)	0.0217 (8)	0.0178 (8)	0.0029 (7)	0.0010 (7)	0.0063 (6)
N13	0.0194 (8)	0.0176 (8)	0.0252 (9)	0.0022 (6)	0.0026 (7)	0.0043 (7)
N14	0.0187 (8)	0.0224 (8)	0.0209 (8)	0.0021 (7)	0.0003 (7)	0.0049 (7)
C7	0.0184 (9)	0.0173 (9)	0.0202 (9)	0.0067 (7)	0.0068 (7)	0.0038 (7)
N17	0.0188 (8)	0.0229 (8)	0.0167 (8)	0.0003 (7)	0.0022 (6)	0.0036 (6)
C3	0.0198 (9)	0.0209 (9)	0.0186 (9)	0.0063 (7)	0.0076 (7)	0.0035 (7)
C5	0.0192 (9)	0.0161 (9)	0.0181 (9)	0.0058 (7)	0.0064 (7)	0.0009 (7)
C1	0.0158 (9)	0.0203 (9)	0.0197 (9)	0.0068 (7)	0.0064 (7)	0.0032 (7)
C6	0.0181 (10)	0.0256 (10)	0.0255 (10)	0.0040 (8)	0.0043 (8)	0.0049 (8)
C4	0.0303 (12)	0.0270 (11)	0.0352 (12)	0.0107 (9)	0.0045 (10)	0.0099 (9)
C2	0.0195 (10)	0.0243 (10)	0.0215 (10)	0.0044 (8)	0.0028 (8)	−0.0003 (8)
C8	0.0304 (12)	0.0301 (11)	0.0216 (10)	0.0045 (9)	0.0096 (9)	0.0083 (8)

### Geometric parameters (Å, °)

**Table:**

S1—O4	1.4743 (14)	N13—H13A	0.8600
S1—O3	1.4766 (14)	N13—H13B	0.8600
S1—O1	1.4795 (14)	N14—C3	1.315 (3)
S1—O2	1.4806 (14)	N14—H14A	0.8600
S2—O5	1.4722 (14)	N14—H14B	0.8600
S2—O6	1.4732 (13)	C7—N17	1.309 (2)
S2—O7	1.4813 (14)	C7—C8	1.493 (3)
S2—O8	1.4822 (14)	N17—H17A	0.8600
N12—C1	1.308 (3)	N17—H17B	0.8600
N12—H12A	0.8600	C3—C4	1.494 (3)
N12—H12B	0.8600	C5—C6	1.487 (3)
N18—C7	1.309 (3)	C1—C2	1.492 (3)
N18—H18A	0.8600	C6—H6A	0.9600
N18—H18B	0.8600	C6—H6B	0.9600
N15—C5	1.308 (3)	C6—H6C	0.9600
N15—H15A	0.8600	C4—H4A	0.9600
N15—H15B	0.8600	C4—H4B	0.9600
N16—C5	1.316 (2)	C4—H4C	0.9600
N16—H16A	0.8600	C2—H2A	0.9600
N16—H16B	0.8600	C2—H2B	0.9600
N11—C1	1.313 (3)	C2—H2C	0.9600
N11—H11A	0.8600	C8—H8A	0.9600
N11—H11B	0.8600	C8—H8B	0.9600
N13—C3	1.303 (3)	C8—H8C	0.9600
			
O4—S1—O3	110.00 (8)	C7—N17—H17A	120.1
O4—S1—O1	110.05 (8)	C7—N17—H17B	119.9
O3—S1—O1	108.83 (8)	H17A—N17—H17B	120.0
O4—S1—O2	109.08 (8)	N13—C3—N14	121.98 (18)
O3—S1—O2	110.04 (8)	N13—C3—C4	119.16 (19)
O1—S1—O2	108.83 (9)	N14—C3—C4	118.87 (19)
O5—S2—O6	110.39 (8)	N15—C5—N16	121.47 (18)
O5—S2—O7	108.65 (8)	N15—C5—C6	119.04 (17)
O6—S2—O7	110.07 (8)	N16—C5—C6	119.49 (17)
O5—S2—O8	109.79 (8)	N12—C1—N11	121.67 (18)
O6—S2—O8	108.87 (8)	N12—C1—C2	119.00 (17)
O7—S2—O8	109.07 (9)	N11—C1—C2	119.31 (17)
C1—N12—H12A	120.1	C5—C6—H6A	109.5
C1—N12—H12B	119.9	C5—C6—H6B	109.5
H12A—N12—H12B	120.0	H6A—C6—H6B	109.5
C7—N18—H18A	120.1	C5—C6—H6C	109.5
C7—N18—H18B	119.8	H6A—C6—H6C	109.5
H18A—N18—H18B	120.1	H6B—C6—H6C	109.5
C5—N15—H15A	120.0	C3—C4—H4A	109.5
C5—N15—H15B	120.1	C3—C4—H4B	109.5
H15A—N15—H15B	119.9	H4A—C4—H4B	109.5
C5—N16—H16A	120.1	C3—C4—H4C	109.5
C5—N16—H16B	119.9	H4A—C4—H4C	109.5
H16A—N16—H16B	120.0	H4B—C4—H4C	109.5
C1—N11—H11A	120.0	C1—C2—H2A	109.5
C1—N11—H11B	120.0	C1—C2—H2B	109.5
H11A—N11—H11B	120.0	H2A—C2—H2B	109.5
C3—N13—H13A	120.1	C1—C2—H2C	109.5
C3—N13—H13B	119.9	H2A—C2—H2C	109.5
H13A—N13—H13B	120.0	H2B—C2—H2C	109.5
C3—N14—H14A	120.0	C7—C8—H8A	109.5
C3—N14—H14B	120.0	C7—C8—H8B	109.5
H14A—N14—H14B	120.0	H8A—C8—H8B	109.5
N18—C7—N17	121.79 (18)	C7—C8—H8C	109.5
N18—C7—C8	119.04 (18)	H8A—C8—H8C	109.5
N17—C7—C8	119.17 (18)	H8B—C8—H8C	109.5

### Hydrogen-bond geometry (Å, °)

**Table:**

*D*—H···*A*	*D*—H	H···*A*	*D*···*A*	*D*—H···*A*
N12—H12A···O1^i^	0.86	2.02	2.838 (2)	158
N12—H12A···S1^i^	0.86	2.93	3.6038 (17)	136
N12—H12B···O6^ii^	0.86	1.99	2.843 (2)	172
N12—H12B···S2^ii^	0.86	2.98	3.7523 (17)	150
N18—H18A···O8	0.86	1.99	2.826 (2)	164
N18—H18A···S2	0.86	2.90	3.6006 (17)	140
N18—H18B···O3	0.86	1.99	2.823 (2)	164
N15—H15A···O2	0.86	2.03	2.841 (2)	157
N15—H15A···S1	0.86	2.92	3.5841 (17)	136
N15—H15B···S2	0.86	3.01	3.7829 (17)	150
N15—H15B···O5	0.86	1.97	2.817 (2)	169
N16—H16A···O1	0.86	2.09	2.915 (2)	160
N16—H16A···S1	0.86	2.85	3.5245 (18)	137
N16—H16B···O3^iii^	0.86	2.00	2.852 (2)	170
N16—H16B···S1^iii^	0.86	2.90	3.6821 (17)	152
N11—H11A···O2^i^	0.86	2.10	2.922 (2)	161
N11—H11A···S1^i^	0.86	2.84	3.5335 (17)	138
N11—H11B···O4^iv^	0.86	2.01	2.856 (2)	170
N11—H11B···S1^iv^	0.86	2.88	3.6657 (17)	152
N13—H13A···O7^ii^	0.86	1.99	2.826 (2)	165
N13—H13A···S2^ii^	0.86	2.93	3.6408 (18)	142
N13—H13B···O4^i^	0.86	1.99	2.835 (2)	165
N14—H14A···O8^ii^	0.86	2.09	2.938 (2)	167
N14—H14A···S2^ii^	0.86	2.87	3.5920 (18)	143
N14—H14B···O6^iv^	0.86	2.02	2.863 (2)	167
N14—H14B···S2^iv^	0.86	2.96	3.6973 (17)	145
N17—H17A···O7	0.86	2.12	2.964 (2)	167
N17—H17B···S2^v^	0.86	2.95	3.7158 (17)	149
N17—H17B···O5^v^	0.86	2.01	2.861 (2)	168
N17—H17B···S2^v^	0.86	2.95	3.7158 (17)	149

Symmetry codes: (i) *x*−1, *y*, *z*; (ii) *x*, *y*+1, *z*; (iii) −*x*+1, −*y*+1, −*z*+1; (iv) −*x*+1, −*y*+1, −*z*+2; (v) −*x*, −*y*, −*z*+1.
